# The Molecular Aspects of Disturbed Platelet Activation through ADP/P2Y_12_ Pathway in Multiple Sclerosis

**DOI:** 10.3390/ijms22126572

**Published:** 2021-06-18

**Authors:** Angela Dziedzic, Elzbieta Miller, Joanna Saluk-Bijak, Marta Niwald, Michal Bijak

**Affiliations:** 1 Department of General Biochemistry, Faculty of Biology and Environmental Protection, University of Lodz, Pomorska 141/143, 90-236 Lodz, Poland; angela.dziedzic@edu.uni.lodz.pl; 2 Department of Neurological Rehabilitation, Medical University of Lodz, Milionowa 14, 93-113 Lodz, Poland; elzbieta.dorota.miller@umed.lodz.pl (E.M.); marta.niwald@umed.lodz.pl (M.N.); 3 Biohazard Prevention Centre, Faculty of Biology and Environmental Protection, University of Lodz, Pomorska 141/143, 90-236 Lodz, Poland; michal.bijak@biol.uni.lodz.pl

**Keywords:** P2Y_12_ receptor, adenosine diphosphate, blood platelets, multiple sclerosis

## Abstract

Epidemiological studies confirm a high risk of ischemic events in secondary-progressive multiple sclerosis (SP MS) patients, directly associated with an increased level of pro-thrombotic activity of platelets. Our work aimed to verify potential molecular abnormalities of the platelet P2Y_12_ receptor expression and functionality as a cause of an increased risk of thromboembolism observed in the course of MS. We have demonstrated an enhanced platelet reactivity in response to adenosine diphosphate (ADP) in SP MS relative to controls. We have also shown an increased mRNA expression for the *P2RY12* gene in both platelets and megakaryocytes, as well as enhanced density of these receptors on the platelet surface. We postulate that one of the reasons for the elevated risk of ischemic events observed in MS may be a genetically or phenotypically reinforced expression of the platelet P2Y_12_ receptor. In order to analyze the effect of the PAR1 (protease activated receptor type 1) signaling pathway on the expression level of P2Y_12_, we also analyzed the correlation parameters between P2Y_12_ expression and the markers of platelet activation in MS induced by selective PAR1 agonist (thrombin receptor activating peptide-6, TRAP-6). Identifying the molecular base responsible for the enlarged pro-thrombotic activity of platelets in SP MS could contribute to the implementation of prevention and targeted treatment, reducing the development of cardiovascular disorders in the course of the disease.

## 1. Introduction

Blood platelets belong to principal hemostatic cells, but besides participating in thrombotic mechanisms, they also play a key role in inflammation, including neuroinflammatory events. Platelets are involved in the pathophysiology of central nervous system (CNS) disorders, for example the pathogenesis of multiple sclerosis (MS), although their role in this pathology still needs to be clarified [1]. The same molecular mechanisms that underlie the hemostatic function of platelets also facilitate the participation of these cells in other physiological and pathological processes due to the cooperation with immunologically competent cells in inflammatory and immune responses [2]. As mentioned, blood platelets serve a critical function in hemostasis, but their small size keeps them in the external blood stream close to the vessel wall and allows them to quickly reach the site of endothelial inflammatory damage, while their enormous reactivity in response to various stimuli make them an important element of the immune response [3]. Platelets, in the physiological condition, are in a resting state in circulation, but become activated due to the action of soluble platelet agonists such as thrombin, ADP, thromboxane A2 (TXA2), and others. The expression of multiple membrane receptors determines the dynamic process of platelet activation. Numerous active molecules induce signal transduction via their respective receptors and intermediate signaling events [4]. The presence of constitutive receptors involves multiple feedback loops and induces activation-dependent receptors, which, like P-selectin, support cross-talk between platelets and endothelial and immune cells [5]. Cell–cell interactions provide crucial mechanisms used by platelets to link thrombosis, inflammation, and related processes, such as diapedesis and infiltration of leukocyte into the affected vessel [6,7,8]. The direct interaction of platelets with endothelium and inflammatory cells using surface receptors promotes leukocyte recruitment to the inflamed tissue [9]. The pathomechanism of MS involves a disruption of the blood–brain barrier (BBB), which is induced by the enormous influx of inflammatory cells. Their recruitment, and in consequence, a pro-inflammatory stimulation of microglial cells, results in the destruction of the myelin sheath, accelerating the formation of demyelinating lesions [10].

MS is a chronic, neurodegenerative, immune-mediated disease, which is considered a complex neurological disorder with diverse pathophysiological mechanism and variable clinical courses, which are not equally represented in the population of patients but may selectively predominate in individuals. There is a large number of various pathogenic effectors, which can be responsible for the neuronal/axonal degeneration in MS [11]. Therefore, MS is unpredictable as well as difficult to treat. The currently used therapies are mainly based on eliminating adverse symptoms accompanying the course of the disease [12].

Determination of molecular mechanisms of increased platelet activity in MS may be beneficial for the improvement of applied treatments. Many reports have proven that blood platelets are chronically overactivated in neurodegenerative diseases, including MS [13,14,15,16]. The vast majority of data on the mechanisms of the platelet pathophysiology of MS is based on research conducted among patients in the first stage of the disease, with a relapsing-remitting MS (RR MS) [16,17,18]. Ultimately, after 10–15 years of disease duration, RR MS in approximately 85% of cases converts into a secondary-progressive (SP MS) disease in which patients suffer irreversible disability progression, without a remission [19]. In contrast to other studies, for many years, our research has focused on disturbances in the homeostatic function of blood platelets exclusively in the SP MS, which is characterized by a particularly high risk of cardiovascular disorders compared to other MS types [20,21]. Despite the fact that SP MS is mainly dominated by neurodegeneration, it is also characterized by an extensive inflammatory process. Many of MS research mainly focuses only on the active phase of the disease (RRMS) and there is still lack of research in the group of patients with progressive phase of MS [22]. Our previous studies not only indicated an excessive intravascular activation of these cells, but also their hyper-responsiveness to the number of physiological agonists in SP MS [23,24]. When considering the pro-thrombotic and pro-inflammatory potential of platelets, it may be highly valuable to recognize the factors potentially predisposing them to overactivation in MS. Our research is focused on seeking the cause of the heightened pro-thrombotic potential of blood in SP MS, as a consequence of molecular changes within blood platelets and/or their precursor cells—megakaryocytes. Shankar et al. have demonstrated that the P2Y_12_ receptor-mediated signaling pathway plays an important role in arachidonic acid release and thromboxane A2 (TXA2) generation in response to thrombin receptor activation. Our recently published work indicates the molecular aspects of an increased PAR1-dependent activation pathway, which may have thromboembolic consequences in the course of MS [25].

The PAR1 signaling pathway is largely mutually dependent on the induction of the P2Y_12_/Gi pathway by ADP [26]. Induction of PAR1 receptor is primarily responsible for the raised expression of P2Y_12_ mRNA, as well as a total P2Y_12_ cellular protein and its cell surface expression due to the exposure to thrombin [27]. ADP is one of the major platelet agonists. ADP acting synergistically with thrombin are fundamental agents for the formation of a platelet plug that leads to the occlusion of a blood vessel [28]. Therefore, dual antiplatelet therapy based on acetylsalicylic acid and clopidogrel (thromboxane A2 (TXA2)- and P2Y_12_-inhibitor, respectively) is the primary treatment of artery ischemic events [29]. Acetylsalicylic acid (ASA) participates in the arachidonic acid cascade initiated by secondary messengers generated during the activation of G-protein-coupled receptors such as PAR and P2Y_12_ [30], and acts as an inhibitor of the cyclooxygenase 1 and 2 (COX-1 and COX-2), lipoxygenase, and cytochrome P450 monooxygenase (CYP450) pathways [31]. COX-1 is constitutively expressed in blood platelets, as well as in neurons and microglial cells, and plays a key role in the conversion of arachidonic acid to essential cell-signaling eicosanoids, while COX-2 is involved in the later stages of an inflammatory reaction when it starts the development of the immune response [32]. The antiplatelet mechanism of ASA activity is based on acetylation of serine at position 529 in COX-1 and 516 in COX-2 leading to inhibition of TXA2 synthesis in the platelets [33]. In addition, ASA affects the activation and aggregation of platelets, and limits platelet degranulation and the release of growth factors and cytokines [34]. Clinical studies have proven that the pharmacologic blockade of platelet P2Y_12_ receptor with the selective antagonist, such as clopidogrel or ticagrelor, as well as genetic deletion of P2Y_12_, significantly diminished the risk of cardiovascular incidents, indicating the major role of the ADP/P2Y_12_ pathway in ischemic events [35,36,37].

The aim of this work has been to verify the potential molecular abnormalities of platelet P2Y_12_, which may be a contributing factor to the well-established increased risk of ischemic events in SP MS. We hypothesized that the increased ADP-dependent platelet reactivity in SP MS is due to an impaired expression or functionality of the P2Y_12_ receptor. For this purpose, we evaluated the ability of blood platelets to activate in response to the ADP action in whole blood using the double-labelled flow cytometry method. P2Y_12_ functionality in SP MS was determined by the basic markers of platelet activation (the level of platelet–platelet aggregates (PAGs), platelet–leukocyte aggregates (PLAs), platelet-derived microparticles (PMPs), and surface expression of P-selectin), and most importantly, we measured the platelet reactivity index (PRI), which is the value obtained by the ADP-dependent VASP (vasodilator-stimulated phosphoprotein) phosphorylation evaluation assay. We have also visually presented the expression level of the platelet P2Y_12_ surface receptor after ADP stimulation.

Moreover, comparative analysis was performed to evaluate the expression of the *P2RY12* gene and the concentration of the P2Y_12_ receptor molecules in blood platelets and megakaryocytes, which are platelet precursor cells. The difference in the surface density of the P2Y_12_ receptor after ADP stimulation between SP MS and control platelets was also illustrated by the flow cytometric method. Focusing on the molecular aspects of enhanced platelet activation through the ADP/P2Y_12_ pathway, in this paper we also analyze the influence of the PAR1-dependent pathway on P2Y_12_ signaling. The mRNA expression level for *P2RY12* gene and the concentration of P2Y_12_ molecules in platelets (as well as in megakaryocytes) were correlated with the level of basic markers of platelet activation induced by synthetic thrombin receptor activating peptide 6 (TRAP-6), which is a selective PAR1 agonist, referring to the results of our previously published paper [25]. This data was compiled with correlation parameters for the expression of *P2RY12* gene or P2Y_12_ proteins vs. the level of PAGs, PMPs, and P-selectin, determined in SP MS blood after ADP stimulation.

## 2. Results

### 2.1. The Level of Platelets’ Markers of Activation

**Figure 1:**
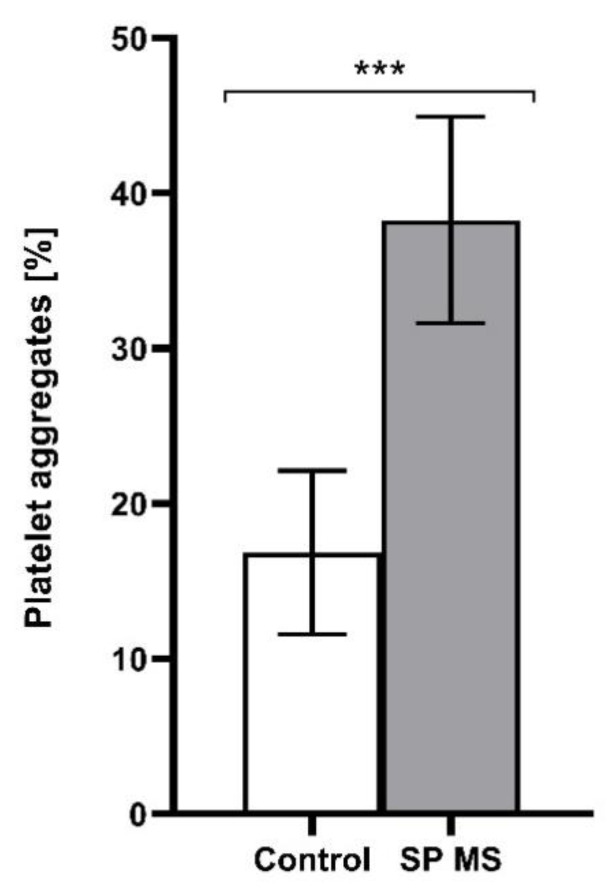
The level of platelet aggregation expressed as a percentage of PAGs relative to the total platelet pool (15,000 CD61-positive objects), measured by flow cytometry method in ADP (20 μM)-treated blood from the secondary-progressive multiple sclerosis (SP MS) (*n* = 55) and control group (*n* = 55). Statistical analysis was performed using the Mann-Whitney U test. The data represents the mean percentage of PAGs ± SD, *** *p* < 0.001.

**Figure 2:**
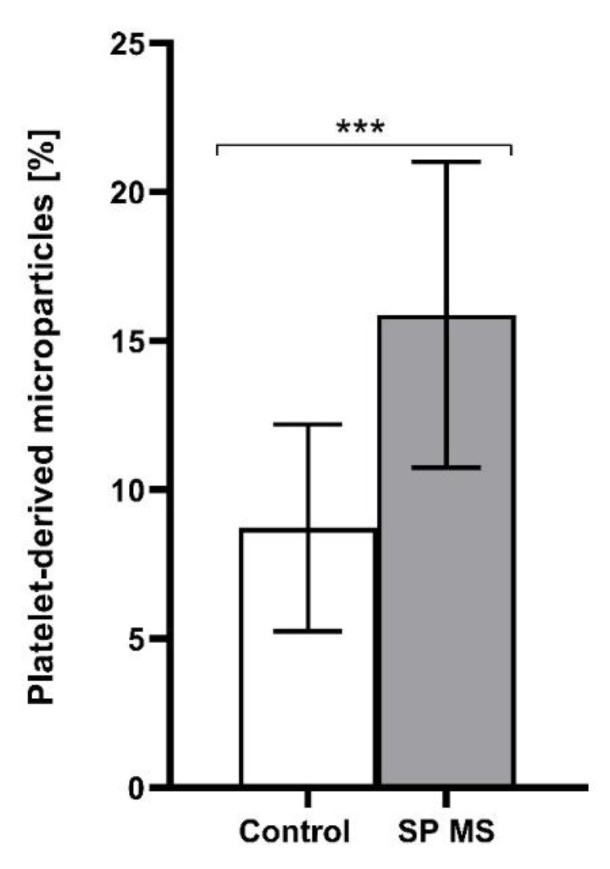
The percentage of PMPs determined relative to the total platelet pool (15,000 CD61-positive objects), measured by flow cytometry method in ADP (20 μM)-treated blood from the secondary-progressive multiple sclerosis (SP MS) (*n* = 55) and control group (*n* = 55). Statistical analysis was performed using the Mann-Whitney U test. The data represents the mean percentage of PMPs ± SD, *** *p* < 0.001.

**Figure 3:**
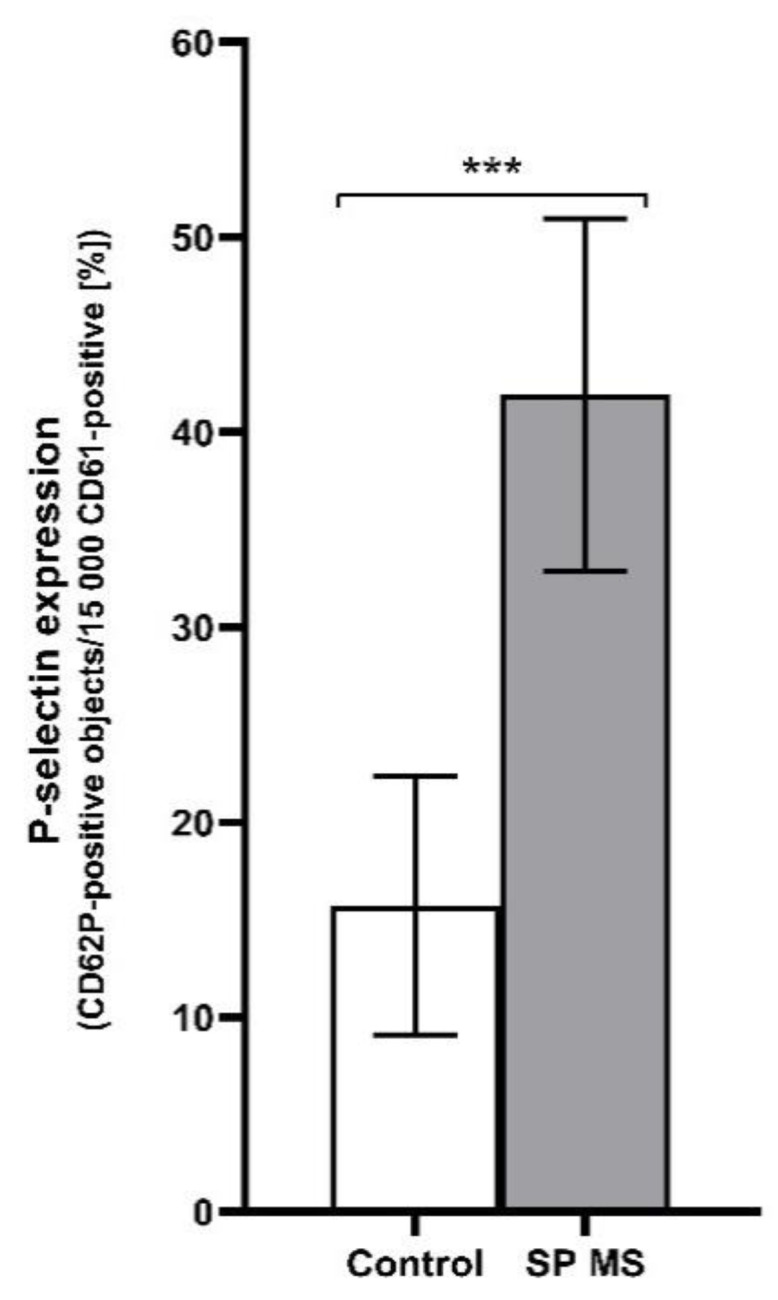
The level of P-selectin expression determined as a percentage of CD61/CD62P-positive objects relative to the total platelet pool (15,000 CD61-positive objects), measured by flow cytometry method in ADP (20 μM)-treated blood from the secondary-progressive multiple sclerosis (SP MS) (*n* = 55) and control group (*n* = 55). Statistical analysis was performed using the Mann-Whitney U test. The data represents the mean percentage of exposed P-selectin ± SD, *** *p* < 0.001.

As a result of our cytometric analysis, we demonstrated an explicit increase in the percentage of PAGs, PLAs, and PMPs, as well as a higher expression of surface P-selectin in SP MS patients compared to control in ADP-stimulated blood platelets (the results for non-stimulated blood platelets was published previously [25]). The examination of blood platelet responsiveness to the action of ADP (20 μM) showed a higher percentage of PAGs (SP MS about 2-fold increase vs. control, *p* < 0.001) (Figure 1) and PMPs (SP MS almost 2-fold increase vs. control, *p* < 0.001) (Figure 2), as well as an elevated surface expression of P-selectin in SP MS platelets (over 2.5-fold vs control, *p* < 0.001) (Figure 3). All results are the percentage expressed per total pool of 15,000 CD61-positive cells (identified as blood platelets).

**Figure 4:**
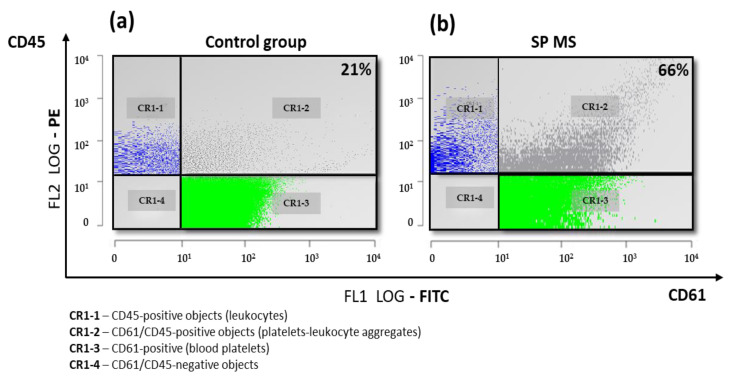
The representative double-fluorescence dot-plots of the pool of PLAs determined as a percentage of CD61/CD45-positive objects relative to the total leukocyte pool (15,000 CD45-positive objects), measured by flow cytometry method in ADP-treated whole blood from control (left dot-plot) (**a**) and secondary-progressive multiple sclerosis (SP MS) (right dot-plot) (**b**). The typical double-fluorescence dot-plots CD61/FITC (FL1) vs. CD45/PE (FL2) represent in log scale the gating strategy for PLAs. Dot-plots are divided into 4 quadrants: CR1-1 (blue dots: CD45-positive objects-leukocytes), CR1-2 (grey dots: CD61/CD45-positive-PLAs), CR1-3 (green dots: CD61-positive objects-platelets), and CR1-4 (represent nonspecific antibody binding, not included in measurement).

The level of PLAs (as a marker of platelet–leukocyte crosstalk mediated by P-selectin) in ADP-stimulated blood from SP MS was also higher in comparison to the control. Figure 4 includes representative double-fluorescence dot-plots of the percentage of formed PLAs (CD61/CD45-positive objects per 15,000 CD45-positive cells) in ADP-treated blood from the control (Figure 4a) and SP MS (Figure 4b).

### 2.2. The Measurement of PRI [%]

**Figure 5:**
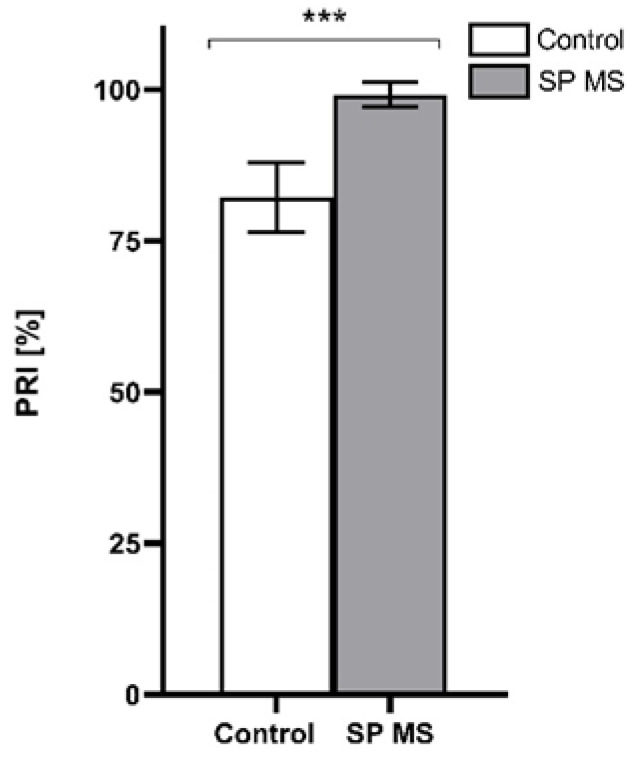
The level of VASP phosphorylation in the analyzed total pool of 15,000 CD61-positive objects after ADP stimulation in the control group (*n* = 55) and secondary-progressive multiple sclerosis (SP MS) patients (*n* = 55), measured by flow cytometry method. Statistical analysis was performed using the Mann-Whitney U test. The data represents the mean percentage of PRI ± SD, *** *p* < 0.001.

In the next step of experiments, we reported a distinctly higher level of the platelet VASP phosphorylation in SP MS patients in comparison to the control group (Figure 5). Blood platelets from almost all blood samples of patients with SP MS reached the maximum level of reactivity in response to ADP (99.8 ± 2%), while the average control PRI was about 20% lower (82.1 ± 5.8%) (*p* < 0.001).

### 2.3. P2Y_12_ Receptor Expression on Blood Platelets [%]

**Figure 6:**
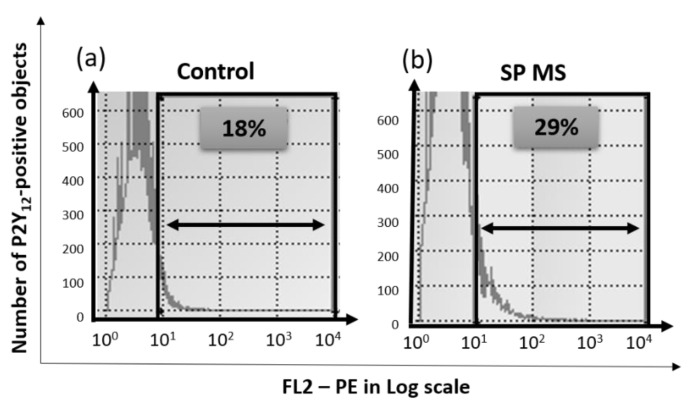
The representative histograms of the expression level of P2Y_12_ receptor determined as a percentage of CD61/P2Y_12_-positive objects relative to the total platelet pool (15,000 CD61-positive objects), measured by flow cytometry method in ADP-treated blood from the control (**a**) and secondary-progressive multiple sclerosis (SP MS) (**b**) groups.

Based on the flow cytometry analysis, the expression level of surface P2Y_12_ receptor as a result of the ADP stimulation was significantly increased in SP MS compared to the control. Figure 6 includes representative histograms for control (a) and SP MS (b).

### 2.4. mRNA Expression for P2RY12 Gene in Blood Platelets and Megakaryocytes

**Figure 7:**
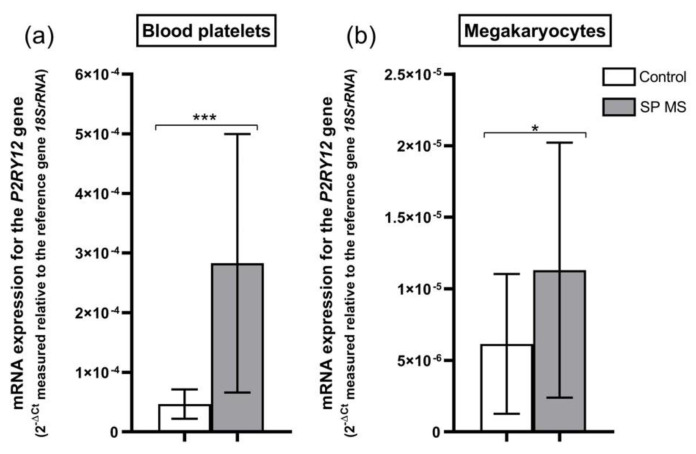
Expression of the *P2RY12* gene (measured at the mRNA level) in platelets (**a**) and megakaryocytes (**b**) from patients with secondary-progressive multiple sclerosis (SP MS) (*n* = 55) and from controls (*n* = 55). Statistical analysis was performed using the Mann-Whitney U test. The results are expressed as the mean value of 2^−ΔCt^ ± SD (according to the reference gene-18SrRNA), * *p* < 0.05, *** *p* < 0.001.

The relative expression of mRNA transcripts for the *P2RY12* gene in blood platelets obtained from patients with SP MS was increased approximately 6-fold (*p* < 0.001) in comparison to the control group (2^−ΔCt^ was 2.82 × 10^−4^ ± 2.16 × 10^−4^ in SP MS vs. 0.47 × 10^−4^ ± 0.27 × 10^−4^ in control) (Figure 7a). Simultaneously, the level of relative expression of mRNA transcripts for the *P2RY12* gene in megakaryocytes was 85% higher in SP MS compared to the control group (2^−ΔCt^ was 1.11 × 10^−5^ ± 0.89 × 10^−5^ in SP MS vs. 0.62 × 10^−5^ ± 0.49 × 10^−5^ in control) (Figure 7b).

### 2.5. Concentration of P2Y_12_ Molecules in Blood Platelets and Megakaryocytes

**Figure 8:**
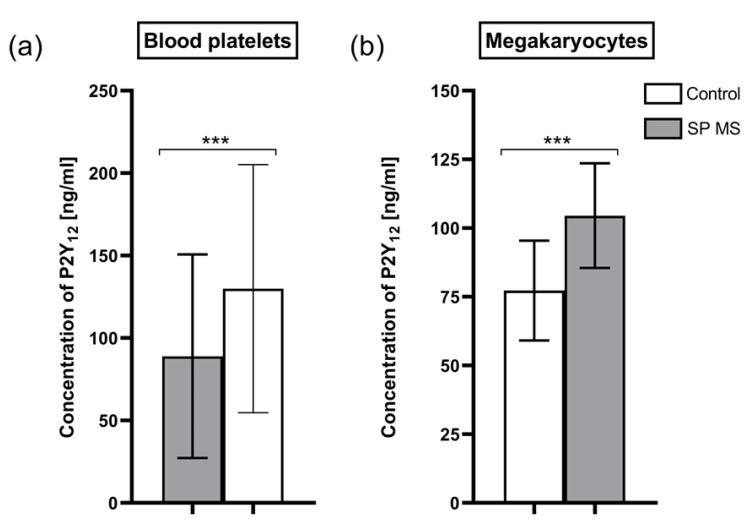
The level of P2Y_12_ (ng/mL) ± SD in blood platelets (**a**) and megakaryocytes (**b**) from secondary-progressive multiple sclerosis (SP MS) patients (*n* = 55) and from control group (*n* = 55). Statistical analysis was performed using the Mann-Whitney U test. The data represents the mean concentration of P2Y_12_ ± SD, *** *p* < 0.001.

Based on the results obtained by the ELISA method, we found that the average concentration of P2Y_12_ molecules in lysates of platelets from patients with SP MS (129.98 ± 75.24 ng/mL) was about 46% higher than the concentration in control platelets (88.93 ± 61.73 ng/mL) (*p* < 0.001) (Figure 8a), while the mean concentration of P2Y_12_ molecules in lysates of megakaryocytes from patients with SP MS was about 35% higher compared to the average concentration in lysates of control megakaryocytes (104.52 ± 19.03 ng/mL vs. 77.28 ± 18.16 ng/mL, respectively) (*p* < 0.001) (Figure 8b).

Additionally, we demonstrated a positive correlation between mRNA expression level for the *P2RY12* gene or the concentration of P2Y_12_ molecules in platelets (as well as in megakaryocytes) and the level of platelet activation markers (PAGs, PMPs, P-selectin surface expression) observed in the blood of SP MS patients after ADP stimulation. Moreover, an analogous statistical analysis was compiled for the mRNA expression level for the *P2RY12* gene or the concentration of P2Y_12_ molecules and the level of platelet activation markers induced via PAR1 stimulated by the TRAP-6 (based on our previously published data [25]) (Table 1).

## 3. Discussion

Blood platelets can be activated by various agonists that bind to specific membrane receptors on the platelet surface. Thus, stimuli such as ADP (thrombin, collagen, and others) activate specific transmembrane receptors that transmit activation signals into the cell. This transduction induces changes in second messenger concentrations, which in turn trigger cellular responses [38].

The purinergic P2Y receptors form the most numerous group of receptors on the blood platelets’ surface: approximately 600 P2Y_12_ and 150 P2Y_1_ receptors per platelet are present [39,40]. These metabotropic receptors contribute separately to the complex process of ADP-induced platelet aggregation; the P2Y_1_ receptor is responsible for mobilization of ionized calcium from internal stores, which initiates aggregation, while the P2Y_12_ receptor supports the gradual, irreversible stage of platelet aggregation by inhibition of ADP-mediated generation of cyclic adenosine monophosphate (cAMP), a negative regulator of platelet activation [41]. Extracellular ADP acts as a direct platelet agonist, but when it is released from platelet storage granules, it acts to expand platelet activation induced by other stimuli [42]. P2Y_12_ is the key ADP receptor that amplifies platelet responses induced by other agonists [43,44]. It has been proven that mice lacking in P2Y_12_ show decreased GPIIb/IIIa activation and reduced adhesion to von Willebrand factor (vWF) [45]. What is more, an irreversible platelet aggregation mediated by both thrombin receptors PAR1 and PAR4 is largely dependent on stimulation of the P2Y_12_/Gi pathway by ADP released from platelets [46,47]. The thrombin is one of the strongest activators of platelets and influences some different signaling pathways. PAR4 forms a dimer with P2Y_12_ receptor by activating the P2Y_12_/PI3K pathway, causing an increase in the stabilization of the formed platelet clot [48,49]. However, according to literature data, PAR1 receptor is mainly responsible for increased expression of P2Y_12_ mRNA, total P2Y_12_ cellular protein, and cell surface expression (on cultured human vascular smooth muscle cells) due to exposure to thrombin [27].

The risk of ischemic incidents such as myocardial infarction and stroke in MS [20,50,51] may raise a question about the level of spontaneous activity and hyperresponsiveness of platelets in this disorder. The pathogenic role of platelets in MS seems to have a multifactorial mechanism. The platelet activation and the role of platelets in hemostatic mechanisms in the further development of the disease in the SP stage have been the subject of our intensive studies for a few years. Our research implies that blood platelet activation in MS could be a phenomenon consequent to the disease’s development, likely as an effect of secondarily endothelial injury, which provides an additional subendothelial stimuli for blood platelet activation [52]. In the previous study, we demonstrated that the level of typical biomarkers of platelet activation was elevated in blood obtained from SP MS patients compared to control [23,25]. The higher baseline level of the activation stage in SP MS platelets has proven a spontaneous activation in vivo. Moreover, we have indicated that blood platelets from SP MS have an increased COX-dependent arachidonic acid metabolism. Higher COX activity leads to increased aggregation and degranulation of platelets and TXA2 synthesis [53]. Furthermore, COX rapidly metabolizes free arachidonic acid to prostaglandin H2 (PGH2), causing the burst of oxygen consumption. Reactive oxygen species (ROS) generation induces harmful lipid peroxidation that is responsible for changes in the permeability of the membrane, and exposition of the platelet receptors, and results in changes in signal transduction [54]. Chronic inflammation and high activity of pro-oxidative processes are the main factors identified as a cause of excessive platelet activation in SP MS.

We evaluated the baseline level of platelet activation immediately after the collection of whole blood, being their natural environment, without platelet isolation, to diminish the risk of triggered activation and creation of artefacts. However, platelet reactivity in response to an action of exogenous stimuli, analogous to physiological agonist, is also a very important parameter specifying the reactivity of platelets ex vivo. Platelet availability in response to the physiological agonists is also a measure of depletion of circulating platelets, which is characteristics of chronic activation. Our current goal has been to follow the ADP-mediated platelet activation pathway. The analysis of blood platelet responsiveness to the action of ADP included measuring the typical activation markers (Figure 1, Figure 2 and Figure 3) and showed the enhanced reactivity of platelets in SP MS. The levels of platelet PAGs, PMPs and P-selectin (CD62P) surface expression were cytometrically determined in whole blood treated with ADP (20 μM). Flow cytometry analysis is a sensitive method routinely used both in diagnostics and in basic research. The measurement in whole blood using flow cytometric assay determines both the activation state of circulating platelets and their reactivity, if it is done with the addition of external platelet agonists. Platelet activation is accompanied by the formation of PAGs and the release of PMPs. PMPs represent approximately 80% of circulating microparticles in the blood of healthy individuals [38], but the research by Giacomazzi et al. showed that increased production of platelet agonists is required to amplify PMPs shedding. In their studies, ADP-triggered PMPs shedding was dose-independent [55]. What is especially interesting, under experimental conditions, is that collagen-induced formation of PMPs in whole blood is dependent on ADP released from red blood cells, and the blockade of platelet ADP receptors, both P2Y_12_ and P2Y_1,_ suppressed collagen-induced PMPs generation [56]. Strong inhibition levels of collagen- and TRAP-6-induced PMPs formation in vitro and their correlation with P2Y_12_ receptor blockade were also observed by Judge et al., reflecting the fact that ADP, acting via P2Y_12_, amplifies the cell responses initiated via the GPVI and PAR1 receptor [57]. Clinical studies confirmed a reduction in PMPs generation in patients with ischemic syndromes treated with clopidogrel, a well-known P2Y_12_ antagonist. Clopidogrel has been shown to provide clinical benefit in coronary syndromes by inhibition of TRAP-6-induced aggregation, pro-coagulant activity (exposure of phosphatidylserine providing a negatively charged pro-coagulant surface), P-selectin expression, and PMPs production [58]. Increased levels of PAGs and PMPs in the peripheral blood indicate not only a higher pro-coagulant but also pro-inflammatory potential in SP MS [59,60,61]. PMPs take part in cellular communication systems, transmitting remote signals from cell to cell, and particularly, in promoting the spread of inflammation [62].

The putative role of activated blood platelets in the development of vessel inflammatory response is due to their ability to adhere to sites of injury, as well as the capacity of platelets to create an aggregates with leukocytes [63]. Platelet secretory responses favor homo- and hetero-aggregates formation as well as heterotypic interactions with, and adhesion to, other vascular, immune, and inflammatory cells [64]. The cell–cell interaction initiates critical mechanisms by which platelets are able to cross-link inflammation and thrombosis [65]. During activation, blood platelets expose a vast number of membrane receptors, and release signaling molecules and soluble mediators from their granules inducing recruitment and activation of additional platelets, resulting in faster clot formation [66,67]. The releasing of biologically active compounds from platelet granules is an extremely important phenomenon, which may induce pro-inflammatory conditions in surroundings of activated platelets. Secreted molecules from granules act in an auto- or paracrine fashion to modulate cell signaling [68]. One of the major biomarkers of the functional status of blood platelets is P-selectin (CD62P), a glycoprotein stored in α-granules of resting platelets. The measurement of CD62P expression on the platelet surface reflects an actual state of the cell degranulation, and for this reason, is a reliable and practical implementation to monitor the activation level of platelets in vivo and in vitro [69]. Our results demonstrated the almost 3-fold higher percentage of P-selectin (*p* < 0.001) exposure after ADP action in whole blood of patients with SP MS, relative to healthy controls (Figure 3). It is particularly noteworthy that despite elevated baseline levels of platelet activation markers in the progressive phase of MS (which was presented in [25]), blood platelets still release large amounts of P-selectin from α granules, and show enhanced reactivity after ADP stimulation, what only demonstrate their strong pro-thrombotic potential. CD62P molecules are among the antigens that are temporarily expressed on the blood platelet surface. and over time, undergo proteolytic cleavage into plasma as a soluble P-selectin (sP-selectin) [70]. It is particularly important because sP-selectin retains its functionality in circulation, and the elevated plasma level of sCD62 is associated with several inflammatory disorders. In our previous work, we have demonstrated higher sP-selectin levels in the plasma of SP MS subjects than in the control group [23]. During the activation of platelets, the CD62P is exposed on their surface, and allows interaction between leukocytes (mainly monocytes and neutrophils) via P-selectin glycoprotein ligand-1 (PSGL-1) presented on leukocytes’ surface. Interaction with CD62P/PSGL-1 has a crucial role in leukocyte rolling and contributes to leukocyte recruitment and extravasation, which enables the link between PLAs and dysfunctional endothelium, which is the key element in blood vessels prone to inflammation development [71]. Selective recruitment of leukocytes occurs in the inflamed areas of damaged vessels, where proteins of the subendothelial layer are exposed and the level of CD62P is enhanced as a result of the deposition of a large number of active platelets [6]. A study conducted by Dixon et al. showed that CD62P/PSGL-1 interactions enhance monocyte COX-2 expression and increase synthesis of pro-inflammatory prostaglandin E2 (PGE2) through molecular mechanisms including cellular adhesion and cytokine signaling [72]. In addition to CD62P, platelets release from their granules a wide variety of other highly bioactive mediators, such as platelet factor 4 (PF-4), transforming growth factor β (TGF-β), RANTES (Regulated on Activation, Normal T Cell Expressed and Secreted), platelet-derived growth factor (PDGF), proteases, and interleukin 1β (IL-1β), which, following release from granules, can participate in platelet aggregation, thrombosis, platelet adhesive interactions with leukocytes, and regulation of cell proliferation [73].

We have shown that the level of PLAs in ADP-stimulated blood from SP MS was higher in comparison to the control, which is presented visually on representative dot-plots in Figure 4. It is known that after the formation of PLAs, the production of pro-inflammatory molecules, such as cytokines and leukotrienes is triggered [69]. Platelet–leukocyte interaction occurring as a consequence of platelet activation plays a key role in the deposition of activated platelets in demyelinating lesions in MS, which leads to brain neurodegeneration [74]. It is believed that the expression of CD62P on the platelet surface fully reflects cell degranulation, while the measurement of its level is a useful tool to monitor the activation status of platelets in vitro and in vivo [75]. A relatively recent study by Habets et al. emphasized the role of platelets in the course of rheumatoid arthritis, an autoimmune disease accompanied by inflammation, analogous to MS. The authors demonstrated an increased expression of P-selectin and elevated platelet aggregation, and most notably, positive correlation between the activation of platelets and disease activity in rheumatoid arthritis [76]. As current knowledge about the mechanisms underlying pathogenesis of MS is still fragmentary, it should be noted that the mutual cellular interactions between platelets, immune cells, and endothelium are closely related to the activation of all types of these cells, which can have serious consequences for maintaining the tightness of BBB and further development of local inflammatory response in CNS [77].

Thus, the increased reactivity of platelets in response to the action of ADP released under pathological conditions in MS may result in the amplification of pro-thrombotic and pro-inflammatory events, also initially triggered by other agonists. We also confirmed a high blood platelet responsiveness to the action of ADP in a flow cytometry diagnostic test based on the VASP phosphorylation level assessment that negatively correlates with the degree of platelet activation in a pathway mediated by the P2Y_12_ receptor. VASP is an intracellular platelet protein, which belongs to profilin or actin-binding proteins, and is involved in cytoskeleton reorganization, adhesion, and platelet aggregation. VASP phosphorylation is upregulated by the cAMP cascade, and ADP inhibits this process through P2Y_12_ receptors. It can, therefore, be proposed that the phosphorylated state of VASP is a specific intracellular marker of P2Y_12_ receptor reactivity, because the low level of the phosphorylated form of VASP is correlated with increased P2Y_12_ receptor activity [78]. The results of VASP phosphorylation are presented as a percentage value of PRI. Our analysis established that PRI reached almost the maximum level (99.1 ± 2%) in all samples from SP MS patients, while the average control platelet reactivity was about 20% lower (Figure 5).

In this work, we have demonstrated that the level of surface P2Y_12_ receptor expression, measured cytometrically as a result of ADP stimulation, was significantly increased in SP MS compared to the control, which is presented visually in Figure 6. What is more, to verify potential genetic and proteomic abnormalities of the platelet P2Y_12_ receptor, which could be a potential cause of its increased ADP-induced activity, we performed a comparative analysis of P2Y_12_ expression both at the mRNA level and in the protein concentration of this receptor’s molecules. In recent years, it has been shown that in blood platelets, despite the lack of a cell nucleus, protein synthesis takes place based on mRNA derived from megakaryocytes, the giant bone marrow cells that are precursors of platelets [79,80], while other platelet proteins are synthesized in megakaryocytes and flow from mature megakaryocytes to the constituted platelets [81]. Therefore, the recognition of the genetic factors responsible for molecular changes of platelet proteome should be defined both at the stage of platelets and megakaryocytes. Following this thought, we performed multidirectional analysis to find the molecular changes at the mRNA and protein levels in both platelets and megakaryocytes. For the first time, we found that mRNA expression for the *P2RY12* gene was enhanced in both platelets and megakaryocytes of SP MS patients (Figure 7), and this was reflected in the increased platelet P2Y_12_ copy number (Figure 8). Considering our results obtained for megakaryocytes, we can suppose that the changes in *P2RY12* mRNA expression observed in SP MS platelets may already occur in precursor cells, before being phenotypically revealed in platelets.

Focusing on the molecular aspects of enhanced platelet activation through the ADP/P2Y_12_ pathway, we also analyzed the influence of the PAR1-dependent pathway on P2Y_12_ signaling. Due to the established association between the PAR1 signaling pathway and cellular expression of P2Y_12_, the level of mRNA for the *P2RY12* gene and the concentration of P2Y_12_ platelet molecules were correlated with the level of typical markers of platelet activation induced by TRAP-6, a selective PAR1 agonist, referring to the results of our previously published paper [25]. This data was compiled with correlation parameters for expression of the *P2RY12* gene or P2Y_12_ proteins vs. the level of PAGs, PMPs, and P-selectin, determined in SP MS blood after ADP and TRAP-6 stimulation. Our analysis showed for the first time that the relative mRNA transcript expression for the *P2RY12* gene and the concentration of P2Y_12_ proteins are significantly correlated with all parameters of platelet activation, both ADP- and TRAP-6-induced (Table 1). Positive correlations between P2Y_12_ cellular expression and the level of activation markers formed analogously to the exposure of platelets to thrombin may provide evidence for the influence of the PAR1 receptor pathway on ADP/P2Y_12_ signaling.

## 4. Materials and Methods

### 4.1. Cytometric Measurements

The level of classic markers of platelet activation, viz. formation of PAGs, PMPs, and PLAs, as well as the P2Y_12_ and P-selectin (CD62P) surface expression, were evaluated using flow cytometry analysis. The fresh whole blood samples, activated with ADP (20 µM, 10 min, 37 °C) (Chrono-Log Corp., Havertown, PA, USA) or TRAP (500 µM, 10 min, 37 °C) (Sigma-Aldrich, St. Louis, MO, USA) or without agonist, were fixed in 1% Cellfix (Becton Dickinson, San Diego, CA, USA) solution for 1 h at 37 °C, and stained with anti-CD61/FITC or anti-CD61/BB515, and additionally with anti-CD62P/PE, P2Y_12_/PE, or anti-CD45/PE antibodies (30 min. in dark, 25 °C). Flow cytometry antibodies: CD61/FITC, anti-CD61/BB515, anti-CD62P/PE and anti-CD45/PE were purchased from BD Biosciences (San Jose, CA, USA), while P2Y_12_/PE antibody was from BioLegend (San Diego, CA, USA). Against the analysis, the prepared samples were dissolved in 0.9% NaCl (20:1) and vortexed. Fluorescence of 15,000 events (CD61- or CD45-positive objects, platelets, and leukocytes, respectively) was measured each time. Gates for PE and FITC fluorescents were assessed based on the fluorescence of unstained probes. All CD61-positive objects with a parameter FSC below 10^2^ were characterized as PMPs, and those above 10^3^ as PAGs, while CD61/CD45-positive objects were recognized as PLAs. Surface expression of P-selectin or P2Y_12_ on CD61-positive cells was determined by fluorescence of CD62/PE or P2Y_12_/PE, respectively. The percentage of CD62P-positive platelets was calculated relative to the total pool of platelets in each sample (15,000 CD61-positive objects), while the level of CD61/CD45-positive objects (PLAs) was calculated relative to the total pool of leukocytes (15,000 CD45-positive objects) measured in each sample. All data analyses were performed in Cube 6 flow cytometry with CyView Software v 1.5.5.8 (Sysmex Partec, Görlitz, Germany).

### 4.2. Assessment of PRI Using the VASP/P2Y_12_ Assay

To monitor a specific response of blood platelets to ADP, we used the VASP/P2Y_12_ flow cytometric kit (BioCytex, Marseille, France) [82]. Under the study conditions, the phosphorylation of VASP correlates with the inhibition of the P2Y_12_ receptor, whereas in the non-phosphorylated state, it is associated with the active form of the P2Y_12_ receptor. The blood samples are incubated with prostaglandin E1 (PGE1) alone, or with PGE1 combined with ADP. After cellular permeabilization, phosphorylated VASP is immunolabeling using the specific monoclonal antibody (anti-VASP-P). Application of double-labelled flow cytometry analysis allows the comparison of two tested conditions and evaluation of the capability of ADP to inhibit VASP phosphorylation in each sample. The PRI was calculated using the correct mean fluorescence intensities (MFIc) in the presence of PGE1 alone or PGE1 with ADP simultaneously, according to the manufacturer’s protocol. The PRI is calculated by using the following calculation: PRI [%] = [(MFIc PGE1 − MFIc PGE1 + ADP)/MFIc PGE1] × 100. The physiological value of the PRI in the control group should be higher than 70% [83].

### 4.3. Isolation of Blood Platelets

From whole blood samples using differential centrifugation (1200 rpmi, 12 min, 25 °C), a platelet-rich plasma (PRP) was obtained. To remove erythrocyte/leukocyte contamination, the PRP was purified using nano-sized MicroBeads conjugated, respectively, to monoclonal human antibodies CD235a and CD45 (Miltenyi Biotec, Bergisch Gladbach, Germany). Then, the PRP was loaded in the appropriate amount onto a MACS MS Column, which was placed in the magnetic field of a MiniMACS Separator. The magnetically labelled CD235a-positive and CD45-positive cells were retained on the column, and free-flowing purified blood platelets were collected in a sterile tube. The quantity of platelets was established photometrically [84], and the final concentration of the platelet suspension was each time diluted to 2 × 10^8^ platelets/mL. The platelet suspension was divided into two equal portions; into one part, the RNA*Later* solution (Invitrogen, Carlsbad, CA, USA) was added, and into the second part, cell lysing buffer (2 M thiourea, 7 M urea, 4% CHAPS, 30 mM Tris) (Sigma-Aldrich, St. Louis, MO, USA) was added. They were then immediately frozen (−80 °C) for further transcriptomic and proteomic analysis.

### 4.4. Isolation of Megakaryocytes

At first, the peripheral blood mononuclear cells (PBMCs) were isolated by density gradient centrifugation. Fresh whole blood was layered in portions (1:1 ratio) onto Gradisol G (Aqua-Med, Lodz, Poland), and centrifuged (2000 rpmi, 30 min, 25 °C). Next, the buffy coat layer with PBMCs fraction was carefully collected, washed twice using PBS (pH 7.4) (Biosigma, Venice, Italy), and centrifuged (1600 rpmi, 12 min, 25 °C). Obtained PBMCs sediment was suspended in reaction buffer (pH 7.4) (2 mM EDTA, 0.5% BSA, PBS pH 7.4). The second step is a magnetic separation, in which the CD61-positive PBMCs (megakaryocytes) were firstly labelled with anti-CD61 MicroBeads. Subsequently, the cell suspension was loaded onto a MACS Column placed in the magnetic field of a MACS Separator. The megakaryocytes retained on the column were flushed out together with applied reaction buffer by strongly pushing the plunger in the column. Finally, megakaryocyte suspension was divided into two equal portions; into one part, the RNA*Later* solution (Invitrogen, Carlsbad, CA, USA) was added, and into the second part, cell lysing buffer (2 M thiourea, 7 M urea, 4% CHAPS, 30 mM Tris) (Sigma-Aldrich, St. Louis, MO, USA) was added. The samples were then immediately frozen (−80 °C) for further transcriptomic and proteomic analysis.

### 4.5. Isolation of mRNA and cDNA Synthesis

Total RNA was isolated from frozen (−80 °C) blood platelets and megakaryocytes suspended in RNA*Later* solution using the ISOLATE II RNA Mini Kit (Bioline, London, UK), which contains a set of reagents that permit inactivation of RNases while stabilizing RNA and allows complete separation from proteins and DNA. Total RNA purity and concentration were measured by comparing the absorbance at 260 and 280 nm, and then samples of total RNA were stored at −80 °C until use. The RT-PCR reaction was accomplished using the Maxima First Strand cDNA Synthesis Kit for RT-qPCR (Thermo Fisher Scientific, Waltham, MA, United States) according to the manufacturer’s protocol.

### 4.6. Expression of the P2RY12 Gene at the mRNA Level in Blood Platelets and Megakaryocytes

The analysis of the gene expression for the P2Y_12_ molecule was made using TaqMan probes: for the human *P2RY12* gene (Hs01881698_s1), as endogenous control, human *18SrRNA* (Hs99999901_s1) was used (Life Technologies, Carlsbad, CA, USA). Gene expression measurements were made on the Real-Time PCR—The CFX96TM Touch System (Bio-Rad, Hercules, CA, USA) using a TaqMan Universal Master Mix II, no UNG (Life Technologies, Carlsbad, CA, USA). The entire procedure was executed under the manufacturer’s protocol. To calculate the relative expression of studied genes, the equation 2^−ΔCt^ (ΔCt = Ct*_target gene_* − Ct*_18SrRNA_*) was used.

### 4.7. Concentration of P2Y_12_ Receptor in Blood Platelets and Megakaryocytes

The level of P2Y_12_ receptor was measured in blood platelets and megakaryocytes using the commercial Human P2Y_12_ ELISA Kit (Fine Test, Wuhan, China) according to the manufacturer’s protocol. All measurements were made using MaxiSorp plates (Nunc, Roskilde, Denmark). Absorbance was measured at 450 nm using the SPECTROStar Nano Microplate Reader (BMG Labtech, Ortenberg, Germany). The concentration of P2Y_12_ in blood platelet and megakaryocyte was determined based on a standard curve expressed as ng/mL.

### 4.8. Statistical Analysis

All results were expressed as mean ± SD. The statistical analyses were performed using Sigma Plot version 14.0 (Systat Software, Inc., San Jose, CA, USA). The results obtained were firstly analyzed for normal distribution with the Shapiro–Wilk test. The significance of the differences between the values obtained from patients with SP MS and controls were analyzed using the unpaired Student’s t-test (for normal distribution) or the Mann–Whitney U test (for non-normal distribution). Spearman’s rank correlation was used for interdependence analysis between P2Y_12_ expression and platelet activation markers. Multiple comparisons were performed using Benjamini–Hochberg, Holm–Sidak, and Bonferroni–Sidak corrections. Throughout the analysis, a level of *p* < 0.05 was considered statistically significant. All graphs were prepared using GraphPad Prism 8.0. (GraphPad Software, San Diego, CA, USA).

## 5. Conclusions

Defining the complex pathomechanisms of MS and the identification of targets specific to individual pathology is fundamental for the development of therapies. The chronic activation of platelets and their over-reactivity in MS is already proven, but the mechanisms of this pathology still need to be clarified. Much remains to be done in terms of understanding of the molecular basis of specific defects of platelet signaling pathways. Currently, based on our findings, we conclude that targeting the platelet P2Y_12_/ADP pathway may represent a novel therapeutic approach to suppression of the adverse pro-thrombotic consequences in MS. Moreover, due to the molecular studies assessing the cellular level of P2Y_12_ expression, it is possible to predict, in patients with SP MS, the risk of developing the consequences of the platelet prothrombotic phenotype.

## 6. Patients

### Clinical Characteristics of Patients

Blood samples from SP MS patients and the control group were collected into CPDA-1 (citrate phosphate dextrose adenine-1) Sarstedt^®^ tubes (Nümbrecht, Germany), in the morning (8–9 a.m.), in fasting status, and stored under the same conditions, according to one established protocol.

The study samples were obtained from 55 patients (female *n* = 32; male *n* = 23) diagnosed with SP MS. Patients were observed for one year and diagnosed according to the revised McDonald criteria by MRI as the requirement and gold standard for MS diagnosis [85], while secondary progressive course of MS (not active with progression) was ascertained as defined by Lublin et al. [86]. The blood samples from SP MS patients were collected at the Neurological Rehabilitation Division III General Hospital in Lodz, Poland. The SP MS patients were under observation at the Neurorehabilitation Ward for three weeks, during which time they did not receive any immunostimulators, immunomodulators, or hormones. The clinical parameters of enrolled SP MS patients are included in Table 2. The EDSS scale ranges from 0 to 10 (in 0.5 unit increments), representing the level of the patient’s disability [87]. BDI evaluation is one of the most widely used psychometric tests for measuring the severity of depression. It is categorized into four types: normal (≤9), minimal depressive symptomatology (10–15), mild depression (16–31), moderate (32–47), and severe depression (≥47) [88].

Control blood samples were collected from 55 healthy volunteers at the Laboratory Diagnostics Center in Lodz, Poland. The control group was matched by age (48.7 ± 12.2) and gender (female *n* = 32; male *n* = 23). Healthy volunteers were not taking any medications, and they had never been diagnosed with MS or other chronic diseases, coagulation disorders, neurological or hormonal illnesses, nor any chronic inflammatory diseases. Subjects enrolled into both groups did not receive any medicaments which could modulate platelet count or function, or influence platelet activation. Each of the healthy volunteers was subjected to screening (including hematological and biochemical tests) to confirm their medical condition. Clinical characteristics of the control group are included in Table 2.

## Figures and Tables

**Table 1:** Correlation coefficient value obtained for concentration of P2Y_12_ or *P2RY12* mRNA expression and level of platelet activity markers (PAGs, PMPs, and P-selectin) after ADP (20 μM) and TRAP-6 (500 μM) stimulation. The correlation was analyzed using Spearman’s rank correlation (Rho) method. Table consists of Rho with 95% CI, probability of correlation (*p*-value), and adjusted *p*-values for multiple tests by Benjamini–Hochberg (FDR), Holm–Sidak, and Bonferroni–Sidak sequential procedures. Corrected *p*-values below 0.05 are considered as statistically significant and highlighted in bold.

Agonist	Platelet Activity Markers	Spearman’s Rank Correlation		Benjamini-Hochberg FDR (Q = 5%)	Holm-Sidak (α = 0.05)	Bonferroni-Sidak (α = 0.05)
		Rho (95% CI)	*p*-Value	Adjusted *p*-Value 1 #	Adjusted *p*-Value 2 #	Adjusted *p*-Value 3 #
mRNA expression for the *P2RY12* gene in control blood platelets *vs*						
ADP	PAGs [%]	0.286 (0.017–0.516)	**0.0328**	**0.0376**	0.1485	0.5509
	PMPs [%]	0.337 (0.073–0.556)	**0.0112**	**0.0202**	0.1165	0.2369
	P-selectin [%]	0.355 (0.094–0.570)	**0.0073**	**0.0143**	0.0911	0.1613
TRAP-6	PAGs [%]	0.535 (0.207–0.756)	**0.0023**	**0.0076**	**0.0450**	0.0538
	PMPs [%]	0.426 (0.067–0.688)	**0.0188**	**0.0279**	0.1485	0.3659
	P-selectin [%]	0.478 (0.132–0.721)	**0.0074**	**0.0143**	0.0911	0.1633
mRNA expression for the *P2RY12* gene in SP MS blood platelets *vs*						
ADP	PAGs [%]	0.512 (0.249–0.705)	**0.0003**	**0.0038**	0.0069	**0.0072**
	PMPs [%]	0.441 (0.161–0.655)	**0.0024**	**0.0076**	**0.0450**	0.0560
	P-selectin [%]	0.327 (0.028–0.572)	**0.0282**	**0.0338**	0.1485	0.4967
TRAP-6	PAGs [%]	0.419 (0.079–0.673)	**0.0150**	**0.0252**	0.1403	0.3042
	PMPs [%]	0.392 (0.045–0.654)	**0.0243**	**0.0306**	0.1485	0.4459
	P-selectin [%]	0.369 (0.013–0.642)	**0.0375**	**0.0411**	0.1485	0.6004
mRNA expression for the *P2RY12* gene in control megakaryocytes *vs*						
ADP	PAGs [%]	0.348 (0.001–0.621)	**0.0436**	**0.0458**	0.1485	0.6570
	PMPs [%]	0.394 (0.0536–0.652)	**0.0213**	**0.0284**	0.1485	0.4035
	P-selectin [%]	0.469 (0.146–0.702)	**0.0051**	**0.0129**	0.0738	0.1155
TRAP-6	PAGs [%]	0.533 (0.203–0.754)	**0.0024**	**0.0076**	**0.0450**	0.0560
	PMPs [%]	0.545 (0.206–0.755)	**0.0023**	**0.0076**	**0.0450**	0.0538
	P-selectin [%]	0.540 (0.213–0.759)	**0.0021**	**0.0076**	**0.0432**	**0.0492**
mRNA expression for the *P2RY12* gene in SP MS megakaryocytes *vs*						
ADP	PAGs [%]	0.445 (0.075–0.707)	**0.0177**	**0.0279**	0.1485	0.3486
	PMPs [%]	0.577 (0.249–0.786)	**0.0013**	**0.0076**	**0.0282**	**0.0307**
	P-selectin [%]	0.553 (0.216–0.773)	**0.0002**	**0.0038**	**0.0048**	0.0048
TRAP-6	PAGs [%]	0.530 (0.185–0.759)	**0.0037**	**0.0104**	0.0576	0.0851
	PMPs [%]	0.499 (0.144–0.741)	**0.0068**	**0.0143**	0.0911	0.1511
	P-selectin [%]	0.433 (0.059-0.699)	**0.0214**	**0.0284**	0.1485	0.4050
Concentration of P2Y_12_ in control blood platelets *vs*						
ADP	PAGs [%]	0.418 (0.082–0.668)	**0.0139**	**0.0350**	0.1894	0.2853
	PMPs [%]	0.429 (0.097–0.676)	**0.0112**	**0.0314**	0.1870	0.2369
	P-selectin [%]	0.373 (0.029–0.637)	**0.0299**	**0.0424**	0.2354	0.5174
TRAP-6	PAGs [%]	0.379 (0.011–0.657)	**0.0385**	**0.0424**	0.2354	0.6103
	PMPs [%]	0.486 (0.146–0.727)	**0.0060**	**0.0314**	0.1240	0.1345
	P-selectin [%]	0.513 (0.176–0.741)	**0.0038**	**0.0314**	0.0838	0.0873
Concentration of P2Y_12_ in SP MS blood platelets *vs*						
ADP	PAGs [%]	0.312 (0.008–0.564)	**0.0387**	**0.0424**	0.2354	0.6122
	PMPs [%]	0.479 (0.204–0.684)	**0.0010**	**0.0252**	**0.0237**	**0.0237**
	P-selectin [%]	0.317 (0.013–0.567)	**0.0361**	**0.0424**	0.2354	0.5862
TRAP-6	PAGs [%]	0.358 (0.000–0.627)	**0.0441**	**0.0463**	0.2354	0.6612
	PMPs [%]	0.367 (0.016–0.634)	**0.0358**	**0.0424**	0.2354	0.5831
	P-selectin [%]	0.393 (0.041–0.658)	**0.0259**	**0.0424**	0.2354	0.4673
Concentration of P2Y_12_ in control megakaryocytes *vs*						
ADP	PAGs [%]	0.496 (0.139–0.739)	**0.0073**	**0.0314**	0.1426	0.1613
	PMPs [%]	0.425 (0.050–0.695)	**0.0241**	**0.0424**	0.2354	0.4432
	P-selectin [%]	0.475 (0.112–0.726)	**0.0107**	**0.0314**	0.1870	0.2275
TRAP-6	PAGs [%]	0.406 (0.027–0.683)	**0.0320**	**0.0424**	0.2354	0.5418
	PMPs [%]	0.438 (0.066–0.703)	**0.0197**	**0.0384**	0.2333	0.3797
	P-selectin [%]	0.439 (0.066–0.703)	**0.0198**	**0.0384**	0.2333	0.3812
Concentration of P2Y_12_ in SP MS megakaryocytes *vs*						
ADP	PAGs [%]	0.476 (0.115–0.727)	**0.0103**	**0.0314**	0.1870	0.2200
	PMPs [%]	0.472 (0.108–0.724)	**0.0112**	**0.0314**	0.1870	0.2369
	P-selectin [%]	0.473 (0.110–0.725)	**0.0110**	**0.0314**	0.1870	0.2331
TRAP-6	PAGs [%]	0.413 (0.036–0.687)	**0.0288**	**0.0424**	0.2354	0.5041
	PMPs [%]	0.400 (0.021–0.679)	**0.0349**	**0.0424**	0.2354	0.5737
	P-selectin [%]	0.441 (0.069–0.705)	**0.0188**	**0.0384**	0.2333	0.3659

**Table 2:** Clinical characteristics of the control group and SP MS patients with reference ranges; all results are expressed as mean ± SD.

Clinical Characteristics	Reference Range	Control Group (*n* = 55)	SP MS (*n* = 55)
Age (years)	-	48.7 ± 12.2	48.6 ± 12.5
Female (%)	-	58	60
BMI (kg/m^2^)	18.5–24.9	22.31 ± 5.12	21.1 ± 9.7
EDSS (0–10)	0–10	NA	5.5 ± 1.8
BDI (≤9)	9–47	NA	9.6 ± 4.6
Mean disease duration (years)	-	NA	12.3 ± 9.5
TG (mg/dL)	35–150	131.45 ± 79.57	73 ± 61.27
TChol (mg/dL)	115–190	189 ± 44.20	201 ± 399.1
HDL (mg/dL)	>45	60.87 ± 16.71	72 ± 20.4
LDL (mg/dL)	<70	110.80 ± 38.73	90 ± 41.5
LEU (×10^3^/μL)	4–10	6.46 ± 1.53	5.15 ± 2.4
RBC (×10^6^/μL)	3.80–5.40	4.81 ± 0.39	4.41 ± 0.7
PLT (×10^3^/μL)	150–400	258.89 ± 68.10	321.95 ± 42.99
GLU (mg/dL)	70–99	96.69 ± 26.07	84.1 ± 12
CR (mg/dL)	0.50–0.90	85.22 ± 16.80	72.4 ± 13.4
TSH (mIU/mL)	0.27–4.20	1.71 ± 0.80	1.6 ± 0.9
AST (IU/L)	<32	21.09 ± 16.09	26.7 ± 14.07
ALT (U/L)	<33	21.15 ± 13.06	28 ± 12.4
PT (sec)	12–16	12.28 ± 2.29	13.5 ± 2.01
INR (sec)	0.91–1.31	1.01 ± 0.05	1.5 ± 0.04
APTT (sec)	26–40	29.97 ± 2.30	31.2 ± 1.45
FG (mg/dL)	150–450	316.94 ± 58.92	320.25 ± 45.87
ESR (mm/h)	3–12	9.35 ± 8.90	8.45 ± 1.54
CRP (mg/L)	<5	3.15 ± 0.99	12.5 ± 5.5

Abbreviations: ALT—alanine aminotransferase; AST—aspartate aminotransferase; APTT—activated partial thromboplastin time; BDI—Beck’s depression inventory; BMI—body mass index; CR—creatinine; CRP—C-reactive protein; EDSS—expanded disability status scale; ESR—erythrocyte sedimentation rate; FG—fibrinogen; GLU—glucose; HDL—high-density lipoprotein; LDL—low-density lipoprotein; LEU—leukocytes; RBC—red blood cells; PLT—platelets; PT—prothrombin time; TG—triglycerides; TChol—total cholesterol; TSH—thyroid-stimulating hormone; INR—international normalized ratio; IU/L—international units per liter; mIU—milli-international units per liter.

## Data Availability

All data generated or analyzed during this study are included in this published article.
